# Collectanea Medica: Consisting of Anecdotes, Facts, Extracts, Illustrations, &c.

**Published:** 1824-04

**Authors:** 


### COLLECTANEA MEDICA:

CONSISTING OF

### ANECDOTES, FACTS, EXTRACTS, ILLUSTRATIONS, &c.

***Relating to the History or the Art of Medicine, and the Collateral Sciences.***

---

Floriferis, ut apes, in saltibus omnia libant,

Omnia nos, itidem, depascimur aurea dicta.

---

ART. I.—*Extracts from "Medical Jurisprudence."* By J. A. PARIS, M.D. &c. and J. S. M. FONBLANQUE, Esq.

### *Of Real and Apparent Death.*

IF life be defined, that power by which organised beings are enabled to resist the physical and chemical operation of surrounding agents, it follows that death must be marked by the occurrence of those phenomena to which the elective attractions, no longer suspended or controlled, will necessarily give rise: hence putrefaction has been considered, by many authors, as the only certain sign of dissolution. Unfortunately, however, this process of decomposition does not immediately display its agency by visible effects: the countenance has remained unchanged for a considerable time after death, and cases have occurred in which its colour and complexion have not only been preserved, but even heightened. This difference in the celerity with which the body putrefies did not escape the observation of the ancients, and, like every other mysterious occurrence, was attributed by them to divine interposition: we accordingly find that their poets mentioned those who preserved the appearance of freshness after death as favoured persons, who had fallen by the gentle darts of Apollo and Diana. Thus Hecuba declares that Hector, although dead for twelve days, still remains fresh, like one who had died by the hands of Apollo. On the other hand, in certain morbid states of the living frame, so feebly do the powers of life resist the operation of physical agents, that, if the body cannot be said actually to enter into a state of putrefaction, it may at least assume appearances so analogous as to be mistaken for it. The test of death, therefore, must rather be sought for amongst those signs which indicate the quiescence, or cessation, of the functions of life, than from those which manifest the decomposition of the organs by which they are performed: and here, again, it may be imagined that no difficulty or fallacy can occur;—the total cessation of respiration, pulsation, sensation, and all motion, it might be supposed, would indicate, to the least experienced, the departure of life; while the general aspect of the body, its pale and livid hue, the coldness of its surface, and the stiffness of its limbs, we might conclude were signs so palpable and satisfactory, as to defy the possibility of doubt. To the skilful medical practitioner, we apprehend, such signs must ever be unequivocal; but we are not prepared to say that a common observer may not be sometimes deceived by them. In cases of extreme debility, as in the latter stage of fever, and where the patient is confined in vitiated air, the exhaustion may be so considerable as to lend all the appearance of death. Indeed, that such cases have occurred, we have no less a testimony than the philanthropic Howard, who, in his work on Prisons, says, "I have known instances where persons, supposed to be dead of the goal-fever, and brought out for burial, on being washed with cold water, have shown signs of life, and soon afterwards recovered." Hippocrates, in his Epidemics, also mentions the case of a woman, who, being in appearance dead from fever, was recovered by throwing thirty amphoræ of cold water over her body. Diemerbroeck relates the case of a rustic, who, having appeared to die of the plague, discovered, after three days, no signs of respiration; but, on being carried to the grave, recovered, and lived many years afterwards: and Paul Zacchias relates an analogous case, which occurred at the hospital of Santo Spirito at Rome. At a period when the small-pox raged with such epidemic fury, and physicians so greatly aggravated its violence by their stimulating plan of cure, there can be no doubt but that many persons were condemned as dead, who afterwards recovered. Amongst the numerous cases that might be cited in support of this opinion, the following may be considered as well authenticated:—The daughter of Henry Laurens, the first president of the American congress, when an infant, was laid out as dead, in the small-pox; upon which the window of the apartment, that had been carefully closed during the progress of the disease, was thrown open to ventilate the chamber, when the fresh air revived the supposed corpse, and restored her to her family. This circumstance occasioned in the father so powerful a dread of living interment, that he directed by will that his body should be burnt, and enjoined on his children the performance of this wish as a sacred duty.

We can also imagine that women, after the exhaustion consequent upon severe and protracted labours, may lie for some time in a state so like that of death, as to deceive the by-standers. A very extraordinary case of this kind is related in the Journal des Sçavans, Janvier 1749.

Dr. Gordon Smith, in his work on Forensic Medicine, has observed, that in cases of precipitancy or confusion, as in times of public sickness, the living have not unfrequently been mingled with the dead; and that, in warm climates, where speedy interment is more necessary than in temperate and cold countries, persons have even been entombed alive. We feel no hesitation in believing that such an event may be possible; but the very case with which the author illustrates his position is sufficient to convince us that this occurrence would be highly culpable, and could only arise from the most unpardonable inattention. "I was," says Dr. Smith, "an eye-witness of an instance in a celebrated city on the continent, where a poor woman, yet alive, was solemnly ushered to the margin of the grave in broad day, and whose interment would have deliberately taken place, but for the interposition of the by-standers." If the casual observer was thus able to detect the signs of animation, the case is hardly one that should have been adduced to show the difficulty of deciding between real and apparent death. Many other illustrations might be adduced, but it is not our intention to amuse the reader with a relation of those numerous *nugæ canoræ*, that enliven several popular productions on the subject of *trances*, premature interments, and extraordinary resuscitations. The public have always betrayed a morbid curiosity upon the subject, and the stories of persons buried alive have ever found a ready access to our credulity, as well as to our compassion.

Amongst the different anecdotes which have been brought forward in support of the popular belief in the frequency of living interment, and in proof of the fallacy of those signs which are commonly received as the unerring indications of death, we read of numerous instances where the knife of the anatomist has proved the means of resuscitating the supposed corpse. Philippe Peu, the celebrated French accoucheur, relates himself the case of a woman, upon whose supposed corpse he proceeded to perform the cæsarean section, when the first incision betrayed the awful fallacy under which he operated. The history of the unfortunate Vesalius, physician to Philip II. of Spain, furnishes another instance, upon which considerable stress has been laid: upon dissecting a Spanish gentleman, it is said that, on opening the thorax, the heart was found palpitating; for which he was brought before the Inquisition, and would probably have suffered its most severe judgment, had not the king interceded in his behalf, and obtained for him the privilege of expiating his offence by a pilgrimage to the Holy Land.

M. Bruhier also relates a case, on the authority of M. l'Abbé Menon, of a young woman, who was restored by the first incision of the anatomist's scalpel, and lived many years afterwards. With respect to the instance of Vesalius, we would make this general observation, which will probably apply to most of the cases on record:—that the movements, which have been observed on such occasions, are not to be received as demonstrations of life; they merely arise from a degree of muscular irritability, which often lingers for many hours after dissolution, and which, on its apparent cessation, may be even re-excited by the application of galvanic stimuli.

The author of the present chapter had once an opportunity of witnessing a most striking manifestation of the popular feeling to which he has just alluded:—A sailor, who had died suddenly on board a vessel in Mount's Bay, was sent on shore for interment on the same evening. This indecent haste in consigning the yet warm corpse of a human being to the grave, excited a very strong and natural feeling in those to whom the fact was communicated. In a few hours the knowledge of the circumstance became general in the town of Penzance, and imagination (which, in cases that interest the feelings, is always ready to colour each feature with the hue most congenial to the fancy,) soon represented the case as one of living interment, and by midnight the impression had produced so strong an effect upon the credulity of the town, that many hundred persons assembled at the house of the mayor, and insisted upon the disinterment of the body. The author, in his professional capacity, was called upon to accompany the magistrates in the investigation, which was accomplished by torch-light, amidst an immense concourse of people. The body was disinterred, when, it is almost needless to add, that not the slightest mark was observed that could in the least sanction the popular belief so readily adopted, and enthusiastically maintained.

Within the last few years, a singular and unphilosophical work has appeared from the pen of a learned divine, which is well calculated to cherish the public credulity upon the subject under discussion, and to excite many groundless alarms, as well as unjust expectations, respecting the possibility of latent life. The reverend author, it must be confessed, has furnished a practical proof of his talents in his favourite art of resuscitation, by recalling into life the numerous idle tales and superstitious histories that we had hoped had long since been for ever consigned to the "tombs of all the Capulets."

The histories of persons having been buried alive, or recovered after apparent death, are not, however, confined to the annals of modern times: we are informed by Diogenes Laertius, that Empedocles acquired great fame for restoring a woman, supposed to be dead, from a paroxysm of hysteria; and Pliny, in his Natural History, devotes a chapter to the subject, under the title of "*De his qui elati revixerunt;*" in which an interesting case is related of Avicola, whose body was brought out and placed on the funeral pile, the flames of which are said to have resuscitated the unhappy victim, but too late to allow it to be rescued from its powers: but such cases merely go to show that the common observer may be deceived. We feel no hesitation in asserting that it is physiologically impossible for a human being to remain more than a few minutes in such a state of asphyxia, as not to betray some sign by which a medical observer can at once recognise the existence of vitality; for, if the respiration be only suspended for a short interval, we may conclude that life has fled for ever. Of all the acts of animal life, this is by far the most essential and indispensable: *breath* and *life* are very properly considered in the Scriptures as convertible terms; and the same synonym, as far as we know, prevails in every language. However slow and feeble respiration may become by disease, yet it must always be perceptible, provided the naked breast and belly be exposed; for, when the intercostal muscles act, the ribs are elevated, and the sternum is pushed forward; when the diaphragm acts, the abdomen swells. Now this can never escape the attentive eye; and, by looking at the chest and belly, we shall form a safer conclusion than by the popular methods which have been usually adopted, such as the placing a vessel of water on the thorax, in order to judge by the stillness or agitation of the fluid; or holding the surface of a mirror before the mouth, which, by condensing the aqueous vapour of the breath, is supposed to denote the existence of respiration, although too feeble to be recognised in any other way.

> "Lend me a looking-glass:
> If that her breath will mist or stain the stone,
> Why then she lives."—*Lear*, act v. sc. iii.

For the same purpose, light down, or any flocculent substance, from the extreme facility with which it is moved, has been supposed capable of furnishing a similar indication; but the result must not be received as an unequivocal proof; and accordingly Shakspeare, with that knowledge and judgment which so pre-eminently distinguish him, has represented Prince Henry as having been thus deluded, when he carried off the crown from the pillow of Henry the Fourth:

> "By his gates of breath
> There lies a downy feather, which stirs not.
> Did he suspire, that light and weightless down
> Perchance must move."

With respect to the above tests, it may be remarked that an imperceptible current of air may agitate the light down, and thus simulate the effects of respiration; while an exhalation, totally unconnected with that function, may sully the surface of a mirror held before the mouth. On the other hand, we have learnt, from experience, that mirrors have been applied to persons in a state of mere syncope, without being in the least tarnished.

Having thus considered the value of the tests of respiration, we shall proceed to appreciate those which have been considered as furnishing no less certain indications of death. The absence of the circulation, the impossibility of feeling the pulsations of the heart and arteries, have been regarded as infallible means of deciding whether the individual be dead; but it is proved beyond all doubt that a person may live for several hours, without its being possible to perceive the slightest movement in the parts just mentioned. It has been thought also, says Orfila, that an individual was dead when he was cold, and that he still lived if the warmth of the body was preserved: there is, perhaps, no sign of so little value;—the drowned, who may be recalled to life, are usually very cold; whilst in cases of apoplexy, and some other fatal diseases, a certain degree of warmth is preserved even for a long period after death. Stiffness of the body is another sign of death, upon which great reliance has been placed; but, as it sometimes happens that it exists during life, it becomes necessary to point out the difference between the stiffness of death, and that which occurs during life in certain diseases. For the following observations upon this subject we acknowledge ourselves indebted to the judicious treatise of Orfila.

1. Stiffness may be very considerable in a person who has been frozen, who is not yet dead, and who may even be recalled to life. This stiffness cannot be confounded with that which is the inevitable result of death, because it is known that the body has been exposed to the action of severe cold, and, above all, because it is very general. In fact, the skin, breasts, the belly, and all the organs, may possess the same rigidity as the muscles; a circumstance not observable in cadaverous stiffness, in which the muscles alone present any degree of resistance: besides, when the skin of a frozen person is depressed, by pressing forcibly upon it with the finger, a hollow is produced, which is a long time in disappearing. When the position of a frozen limb is changed, a little noise is heard, caused by the rupture of particles of ice contained in the displaced part.

2. The stiffness to which the late M. Nysten has given the name of *convulsive*, and which sometimes manifests itself in violent nervous diseases, may be easily distinguished from *cadaverous* stiffness. When a limb is stiff in consequence of convulsions, &c. the greatest difficulty is experienced in changing its direction, and, when left, it immediately resumes its former position: it is not the same in stiffness from death,—the limb, the direction of which has been changed, does not return to its former position.

3. The stiffness which occurs in certain forms of syncope can never be confounded with cadaverous stiffness; for, in the former case, the stiffness takes place immediately after the commencement of the disease, and the trunk preserves a degree of warmth: whereas, the cadaverous stiffness is not observed until some time after death, and when the heat of the body is no longer evident to the senses.

If, from a cause which it is not always possible to foresee, the individual, who has been thought dead for a long time, be cold and *flexible*, instead of offering a certain degree of stiffness, and at the same time if no evidence of putrefaction has as yet displayed itself, the body ought not to be buried hastily. "Satius est adhiberi millies nimiam diligentiam, quam semel omitti necessariam."

The cadaverous state of the face, of which Hippocrates has given the following description, has been regarded as a sign of real death:—The forehead wrinkled and dry; the eye sunken; the nose pointed, and bordered with a violet or black circle; the temples sunken, hollow, and retired; the ears sticking up; the lips hanging down; the cheeks sunken; the chin wrinkled and hard; the colour of the skin leaden or violet; the hairs of the nose and eyelashes sprinkled with a kind of yellowish white dust. It must be admitted that such signs, if taken separately, are of no value, since they are sometimes observed in patients twenty-four or forty-eight hours before death; while, on the other hand, they are often absent in cases of sudden dissolution.

The softness, dimness, and, above all, the flaccidity of the globe of the eye, have been considered as very unequivocal in their indication. Professor Louis has offered some remarks upon this subject worthy our notice: he says that, in the dead, the transparent cornea is commonly covered with a thin slimy membrane, which breaks in pieces when touched, and is easily removed by wiping the cornea: but he remarks that some appearance of it takes place in the eyes of the dying, and also allows that it may be the result of disease. So much for the value of this sign. The one which follows appears to us no less exceptionable. In a few hours after death, adds this author, the eyes become soft and flabby, an effect not to be produced under any circumstances in the living body: we join in this opinion; but how often does it happen that the globe of the eye undergoes no alteration in form until the putrefactive process has been fully established?

### *Of the Physiological Causes, and Phenomena of Sudden Death.*

It has been asserted by Bichat, that the immediate cause of death, when it takes place suddenly, must be the cessation of the functions of the heart, brain, or lungs; although it is sometimes difficult to determine which of these organs is the first to fail in its action. This may be well exemplified by the poisonous operation of arsenic upon the animal economy, which, when introduced into the circulating system, will, according to the valuable experiments of Mr. Brodie, occasion stupor and paralysis, a feeble and intermitting contraction of the heart, and slow and laborious respiration: but it is found that, in some cases, one order of symptoms will predominate, and be the first to display themselves, whilst in others the very contrary will obtain, without perhaps our being able to assign the immediate cause of such deviations. There are, moreover, cases of sudden death, in which the principle of animation would seem to be at once annihilated in every part of the animal machine, and when every organ appears to be simultaneously affected, as in that occasioned by the agency of intense cold, and sometimes, for it is not in every instance, by that of lightning or electricity. Still, as a general proposition, the aphorism of Bichat must be admitted; and we shall proceed to investigate the subject of sudden death, as connected with medico-judicial inquiry, upon principles deduced from the enlightened views of this distinguished philosopher.

To the able and satisfactory researches of our English physiologist, Mr. Brodie, we are also greatly indebted for a correct notion of the nature, and order of succession, of those events by which life is quickly extinguished. His attention was, many years ago, directed to one important branch of this subject,—to the investigation of that series of changes produced on living bodies by the operation of poisons; the results of which were published in the Philosophical Transactions. Since that period, he has diligently pursued the subject in its more extensive ramifications; and in his Lectures, delivered from the anatomical chair of the College of Surgeons during the last year, he presented a condensed and philosophical history of the phenomena of death in general, in which he elucidated many leading points that were before obscure, established several propositions that have long been considered doubtful, and rejected a mass of popular error, which, under the sanction of authority, has continued to retard our inquiries, and to embarrass and misguide our practice. The author of the present section of this work has to acknowledge the kindness and liberality by which he is enabled to avail himself of these luminous researches, having been furnished by his friend, Mr. Brodie, with the manuscript notes from which the lectures were delivered.

The organs more immediately necessary to life are, the Heart, which conveys to every part of the body that fluid, without a constant supply and change of which vitality must be speedily exhausted; and the Lungs, by whose functions this essential fluid undergoes those unknown changes, from the action of the atmosphere, which adapt it for the performance of the important duties to which we have alluded.

In conformity with these views, the functions of the heart, and their connexion with those of the lungs and brain, very naturally present themselves as the first objects of physiological inquiry; and there is certainly no discovery in modern times more interesting in its relations, and at the same time so useful in practical application, as that which has determined the nature of the connexions between the functions of respiration and the motions of the heart; and shown why the cessation of the former should occasion the destruction of the latter. The existence of this mysterious connexion constituted a subject of interest and inquiry in the more remote ages, and it will not be unprofitable to take a review of the different theories which have been proposed for its explanation. Until the celebrated experiment of Hook, it was supposed that the heart's motion was maintained by the alternate contraction and dilatation of the lungs, in the act of breathing; but the extraordinary philosopher above mentioned decided this point by exposing the thorax of a dog, and separating the pleura extensively from the external surface of the lungs: and then, by means of a pair of double bellows, keeping up a constant stream of air through the air-cells. By this contrivance, respiration was duly performed, while the lungs remained motionless, and yet it was found that the vigour of the heart's action was not in the least impaired: whereas, if the theory which Hook undertook to refute had been founded in truth, the heart, under such circumstances, must necessarily have become quiescent. Mr. Hunter supposed the existence of a sympathy, or association, between the motions of the heart and lungs; and the same opinion appears to have been entertained by Dr. Currie. Dr. Darwin deduced the existence of this immediate connexion from that general law of the animal economy, by which motions that are frequently repeated in succession acquire the power of recurring in the same order, independently of the original exciting cause: "it is thus (says he,) that, by the stimulus of the blood in the right chamber of the heart, the lungs are induced to expand themselves." Dr. Bostock, however, has very satisfactorily opposed this hypothesis, by observing that, in the fœtus, the heart commences its contractions immediately upon its formation, while the lungs remain perfectly at rest; and that, when the animal leaves the uterus, the motion of the lungs commences; but the periods of the contraction of the diaphragm bear no determinate ratio to those of the systole of the heart.

It was long supposed that the cessation of respiration occasioned that of the heart's motion, in consequence of the black blood not having sufficient power to stimulate its fibres; but does not the right side of the heart, which under all circumstances contains de-oxygenated blood, contract with a vigour equal to that of the left? It was reserved for Bichat to offer a true explanation of this phenomenon: he has very justly stated that, in consequence of the suspension of the respiratory function, the coronary vessels, by which the muscular structure of the heart is supplied, are compelled to carry black instead of scarlet blood; a fact which, in itself, is quite adequate to explain the cause of the heart ceasing to contract; for the irritability of this, like that of every other muscle, can be alone maintained by duly oxygenized blood. But it remains to be shown how the functions of the brain and nervous system stand related to those of the heart and lungs. Although the agency of nervous influence is necessarily involved in impenetrable obscurity, yet we shall not have much difficulty in proving that *the brain is immediately necessary to life, only because the muscles of respiration owe their action to its influence.* M. Lallemand has published the history of a fœtus, in which the brain and spinal marrow were equally deficient; notwithstanding which, it even exceeded the usual size, the heart was perfect, and it was evident that the circulation had been properly performed: no sooner, however, was the monster born than it perished, because the diaphragm and other muscles of respiration were unable to perform their functions without the aid of nervous excitement; no air was therefore inhaled into the lungs, and in a few minutes the heart ceased to contract, from the deficient supply of oxygenized blood. If the phrenic nerves of a quadruped be divided, the motion of the diaphragm ceases, and the animal breathes by the motion of the ribs alone, panting and respiring with difficulty and distress. If the spinal marrow be divided below the origin of the phrenic nerves in the lower part of the neck, no interruption is given to the transmission of the nervous influence to the diaphragm, but the ribs now become motionless, and respiration is performed by the diaphragm only. If the spinal marrow be divided in the upper part of the neck, above the origin of the phrenic nerves, the nervous influence is neither transmitted to the diaphragm, nor to the muscles which produce the motion of the ribs, and respiration is entirely suspended. Under these circumstances, the heart continues to contract for some minutes, after which it ceases, as there is no supply of blood which has received the influence of the air; and, consequently, the muscular fibres of the heart lose their excitability, and the blood is no longer circulated. If, however, the lungs be artificially inflated before the action of the heart has ceased, its motions are continued. The experiment may also be very satisfactorily varied in the following manner:—Apply a ligature to the carotid arteries in the neck, so as to prevent the occurrence of hemorrhage, and then decapitate the animal: if respiration be now artificially maintained, the heart will suffer no disturbance in its motions, but the circulation will be preserved for several hours in the body of the decapitated animal. In further illustration of this view of the subject, Mr. Brodie observes, that many reptiles, which are capable of respiring by means of the skin, will survive the loss of the brain for so long a period, that the wound made by decapitation becomes cicatrized, and death only takes place at last in consequence of inanition. *(Manuscript notes.)*

In farther illustration of these views, let us observe the mode in which death takes place in apoplexy, or in cases of pressure on the brain, whether occasioned by a depressed portion of bone, or by blood extravasated within the cranium. At first the patient is insensible to all external impressions, but the breathing is not affected; after an interval, however, the respiration becomes difficult and laborious, and the purple hue of the lips and cheeks, from the sub-cutaneous vessels, demonstrates that the blood is imperfectly oxygenized. The arterial action becomes more slow, in proportion only as the respiration is more difficult; and the pulse may even be distinguished at the wrist after the breathing has altogether ceased. Under such circumstances, it is obvious that life might be protracted for several hours by artificial inflation of the lungs; but, as no ultimate benefit could be derived from such an operation, its expediency may be fairly questioned.

Enough has been said to show that the brain is not *immediately* necessary to the action of the heart; but Mr. Brodie has very justly observed, that the general proposition thus established must not lead us to the conclusion, that the heart is therefore incapable of being affected by violent impressions on the nervous system. The fact is quite otherwise; for, although the brain may be removed, and the circulation be nevertheless maintained by artificial respiration, yet an injury inflicted on the brain, of another kind, may be followed by those immediately fatal consequences, which decapitation itself would not produce. Dr. Wilson Philip states that, if the brain be violently crushed, the action of the heart is immediately stopped; and the fact is too notorious to be questioned, that a blow on the head is frequently succeeded by syncope. There are but few circumstances, says Mr. Brodie, in the history of the animal economy, which appears more remarkable than this fact, that *an injury of a part which is not immediately essential to the heart's action, should nevertheless, under certain circumstances, have the effect of occasioning its immediate cessation.*

The late researches of Le Gallois may, perhaps, receive further elucidation from the above proposition. This physiologist has stated that, if a wire be introduced into the theca vertebralis, and be moved upward and downward, so as to destroy the texture of the spinal marrow, the action of the heart presently ceases; and he from thence advances to the conclusion, not only that the spinal marrow is necessary to the heart's action, but that every part of the animal body derives its vital properties from it. From what I have observed, says Mr. Brodie, *(manuscript notes,)* in the repetition of the foregoing experiment, I should infer that the fact is correctly stated, as far as it relates to warm-blooded animals, but the conclusions are undoubtedly premature; and the history of the fœtus, as related by Lallemand, in which, notwithstanding the absence of the brain and spinal marrow, the child was even larger than usual, the heart perfect, and it was manifest that the circulation had been duly performed, is in direct opposition to such a theory. We must here agree with Mr. Brodie, that such phenomena are quite incompatible with the doctrine in which the spinal marrow is supposed to be directly necessary to the existence of vitality in the system generally, and to the action of the heart in particular; and that we must therefore look for some other explanation of the effects which are produced by the destruction of the spinal marrow in warm-blooded quadrupeds. May they not be explained by supposing them to be the effect of the shock which must necessarily attend the removal of the spinal marrow, which can never be effected with the facility which attends decapitation?

We have deemed it necessary to offer these few remarks upon the relations which subsist between the functions of the heart, lungs, and brain, inasmuch as the propositions which have been thus established respecting them can alone lead to a correct pathology of those diseases by which life is suddenly extinguished, or suggest a rational and effectual plan of treatment in cases of suspended animation.

